# Modified Latarjet with or without remplissage for recurrent anterior shoulder instability: a prospective randomized trial

**DOI:** 10.1016/j.jseint.2026.101696

**Published:** 2026-03-14

**Authors:** Hosam Elsayed Abd-Elzaher, Hatem G. Said, Maher A. Elassal, Mohamed M. Abdel Hamid, Mohamed Kamal El Gafary, Moustafa Kamal El Gafary

**Affiliations:** a Department of Orthopedic Surgery, Azhar Assiut University, Assiut, Egypt; b Department of Orthopedic Surgery, Assiut University, Assiut, Egypt; c Department of Orthopedic Surgery, Helwan University, Cairo, Egypt

**Keywords:** Latarjet procedure, Remplissage technique, Hill-Sachs lesion, Glenoid bone loss, Shoulder instability, Shoulder dislocation, On-track lesions, Off-track lesions

## Abstract

**Background:**

Recurrent anterior shoulder instability with glenoid bone loss remains a surgical challenge. The Latarjet procedure has shown reliable results, yet controversy persists regarding the role of adding remplissage. While some studies suggest it may improve stability in cases with concomitant Hill-Sachs lesions, others report no clear functional advantage and potential restriction of external rotation.

**Methods:**

A prospective randomized, single-blinded trial was conducted from 2016 to 2019, enrolling 60 patients with recurrent anterior shoulder instability, glenoid bone loss >25%, and off-track Hill-Sachs lesions. Patients were randomized to modified Latarjet alone (n = 30) or Latarjet plus remplissage (n = 30). Outcome assessors were blinded, and follow-up was 24 months. Evaluations included range of motion, Rowe, Oxford, Constant scores, visual analog scale pain, satisfaction, return to activity, and complications.

**Results:**

After ≥2 years, all clinical outcomes had improved significantly in both groups (*P* < .01), with no significant differences between the groups. Mean Rowe: 85.67 ± 21.16 vs. 85.17 ± 17.83 (*P* = .92); visual analog scale: 2.93 ± 1.86 vs. 2.73 ± 1.33 (*P* = .63); Constant: 84.36 ± 12.69 vs. 86.56 ± 11.41 (*P* = .48); Oxford: 38.70 ± 9.02 vs. 40.36 ± 9.03 (*P* = .47). Return to preinjury activity: 25/30 vs. 26/30 (*P* = .72). External rotation loss was greater in Group 2 (12.47 ± 1.67° vs. 25.67 ± 6.0°, *P* < .01). Operative time was longer in Group 2 (60.71 ± 12.8 vs. 86.5 ± 12.7 minutes, *P* < .001).

**Conclusion:**

Both techniques provided significant and comparable clinical improvement. The addition of remplissage was associated with more post-operative limitation of external rotation and longer operative times.

The glenohumeral joint has the greatest range of motion (ROM) of any joint in the human body and is also the most frequently dislocated. Anterior shoulder instability accounts for the majority of these dislocations and predominantly affects young, active individuals in their second and third decades of life.^19^^,^^20^^,^^24^ Previous research has shown that significant glenoid bone loss and Hill-Sachs lesions are strongly associated with failure of isolated arthroscopic Bankart repair.^3^

Following the introduction of the glenoid track concept by Itoi et al,^23^ Di Giacomo et al further refined this concept, providing a widely accepted method to guide surgical management of recurrent anterior shoulder instability.^7^^,^^8^ According to this approach, patients with >25% glenoid bone loss and off-track Hill-Sachs lesions remain a surgical challenge, as intraoperative engagement of the Hill-Sachs defect may still occur even after a Latarjet procedure.^8^ A systematic review of 3,211 shoulders recommended bony augmentation procedures that increase the glenoid track as the most effective treatment for anterior shoulder instability in the presence of significant bone loss.^14^

Griesser et al compared arthroscopic Bankart repair with remplissage vs. the Latarjet procedure in patients with large, engaging Hill-Sachs lesions and reported that both approaches were safe and reliable, with low recurrence rates.^10^ Importantly, their cohort underwent labral repair in addition to remplissage, highlighting the combined role of soft tissue and bony procedures.^13^ In contrast, other studies have concluded that the Latarjet procedure alone may adequately reduce the risk of Hill-Sachs engagement and advocate its use as the primary treatment, regardless of the severity of bone loss.^1^^,^^9^^,^^22^^,^^26^^,^^27^

Conversely, Mook et al^16^ investigated predictors of outcomes after Latarjet in patients with combined glenoid and humeral defects, demonstrating that those with off-track Hill-Sachs lesions were 4 times more likely to develop recurrent instability. Additional studies have reported that remplissage can reduce redislocation rates from over 25% to approximately 5% in patients with large Hill-Sachs lesions.^4^^,^^25^ These findings reflect the ongoing controversy regarding whether remplissage should be performed as an adjunct to glenoid reconstruction with Latarjet.

The remplissage technique, originally described by Wolf and Pollack, involves posterior capsulotenodesis of the infraspinatus tendon into the Hill-Sachs defect to prevent engagement.^17^ Despite increasing popularity, its routine addition to the Latarjet remains debated.

The objective of the present study was to compare the clinical outcomes of the modified Latarjet procedure alone vs. the modified Latarjet combined with remplissage in patients with recurrent anterior shoulder dislocation, glenoid bone loss >25%, and off-track Hill-Sachs lesions (Table I).^13^

**Table I:** Algorithm of management using the glenoid track concept.

Recommended treatment	Hill-Sachs interval and glenoid track	Glenoid defect (glenoid bone width)	Group
Arthroscopic Bankart repair	HSI < GT (on track)	<25%	1
Arthroscopic Bankart repair plus remplissage	HSI > GT (off track)	<25%	2
Latarjet procedure	HSI < GT (on track)	≥25%	3
Latarjet procedure with or without humeral-sided procedure (humeral bone graft or remplissage)	HSI > GT (off track)	≥25%	4

*GT*, glenoid track; *HSI*, Hill-Sachs interval.

## Methods

This prospective randomized, single-blinded study was approved by our institutional ethics committee. This study was conducted in accordance with the CONSORT (Consolidated Standards of Reporting Trials) guidelines for randomized clinical trials. *Clinical trial registry: Pan-African Clinical Trial Registry (PACTR201702001986912; Date of Registration: 18/01/2017).*

Patients scheduled for surgery for recurrent anterior shoulder instability between January 2016 and December 2019 were assessed for eligibility according to the following.

Inclusion criteria: recurrent anterior shoulder dislocation (≥3 episodes), glenoid bone loss >25% of the glenoid width on 3-dimensional (3D) CT, off-track Hill-Sachs lesion on 3-dimensional computed tomography (3D CT) (Hill-Sachs interval [HSI] > glenoid track), and minimum follow-up of 2 years after surgery.

Exclusion criteria: isolated soft tissue Bankart without bone loss, multidirectional instability, voluntary dislocation, fracture-dislocation, or associated rotator cuff tear; uncontrolled pre-operative seizures (including epilepsy or tramadol-induced seizures); ipsilateral disabilities such as advanced arthritis, paralytic limb, chronic septic joint, or amputation; patients with incomplete follow-up duration (less than 24 months), revision cases and glenohumeral arthritis.

**Figure 1:**
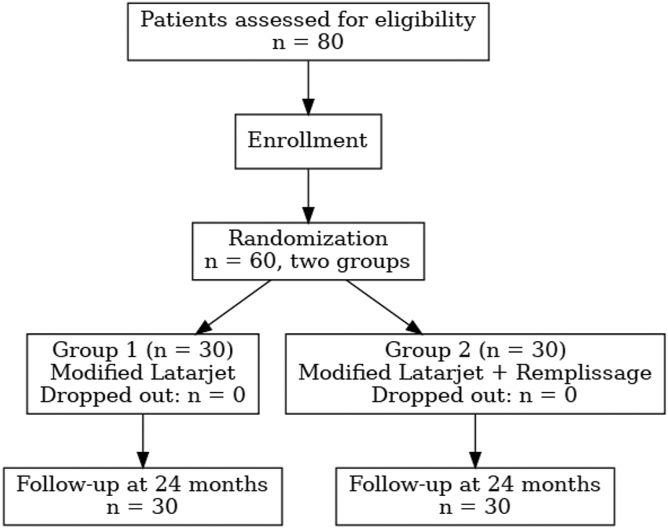
CONSORT-style flow diagram of patient selection, randomization, and follow-up. *CONSORT*, Consolidated Standards of Reporting Trials.

After applying these criteria, 60 patients remained eligible and were classified into 2 groups. Group 1 comprised 30 patients who underwent the modified Latarjet procedure, and Group 2 comprised 30 patients who underwent the modified Latarjet plus remplissage. Randomization was performed by simple randomization Figure 1.

### Blinding

The operating surgeon was blinded to group allocation until the time of surgery. Clinical outcome data (patient-reported outcome measures [PROMs] and ROM assessments) were collected by an independent assessor who was blinded to the participants' group allocation. Study participants themselves and the operating surgeon were not blinded to the procedures performed. The absence of participant blinding is acknowledged as a limitation of the study design.

### Radiological assessment

Pre-operative CT imaging was performed using a 64-channel CT scanner. The scans were reconstructed into humeral-subtracted, three-dimensional en face glenoid views (sagittal oblique). Images were transferred to the Extended Brilliance Workstation (Philips EBW) and reviewed using the hospital's Picture Archiving and Communication System. All radiographic measurements were performed by the senior author (H.A.), a fellowship-trained orthopedic surgeon with more than 10 years of experience in shoulder arthroscopy. To minimize interobserver variability, all measurements were performed by a single observer and repeated on 2 separate occasions to ensure internal consistency. In addition, interobserver and intraobserver reliability were assessed using kappa statistics, with independent verification by a musculoskeletal radiologist (also with >10 years of experience).

**Figure 2:**
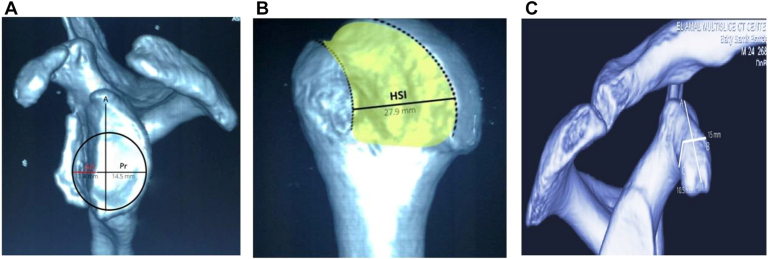
(A) Showing the best-fit circle method. (B) The Hill-Sachs interval. (C) The Coracoid length, width, and thickness. *HSI*, Hill-Sachs interval.

Glenoid measurements were performed using the best-fit circle method.^21^ Parameters included glenoid diameter (GD), glenoid defect (Gd), Gd ratio, and glenoid track (83% of GD–Gd). The HSI was measured on subtracted posterolateral humeral head views (HSI = Hill-Sachs lesion width + bone bridge to the rotator cuff footprint). Coracoid width and thickness were also measured, with 1 mm subtracted to account for decortication during graft preparation demonstrated in Figure 2.

### Surgical technique

**Figure 3:**
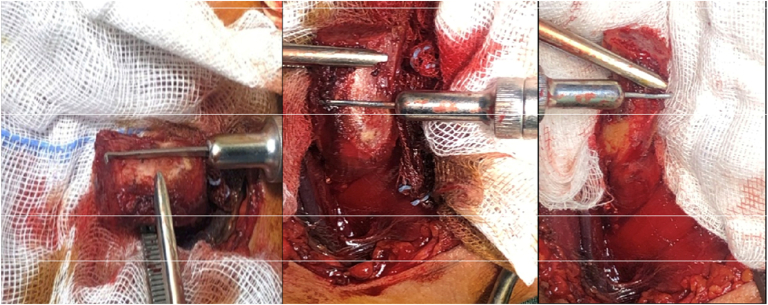
The anthropometric measurements of coracoid length, width, and thickness.

All procedures were performed by the senior author, a fellowship-trained orthopedic surgeon with more than 10 years of experience in shoulder arthroscopy and reconstruction. All cases were operated under general anesthesia in the beach chair position, with careful padding of all bony prominences. A pre-operative examination under anesthesia and confirmatory diagnostic arthroscopy were conducted in every patient. Hill-Sachs engagement was assessed with the arm in 90° of abduction and progressive external rotation before joint distension, and glenoid bone loss was confirmed arthroscopically through the anterosuperior portal. Both the isolated Latarjet and the Latarjet plus remplissage procedures were performed without traction. The remplissage was performed using the technique described by Purchase et al,^17^ while the congruent-arc Latarjet was performed according to the method of de Beer et al.^6^ Anthropometric parameters of the coracoid (length, width, and thickness) were evaluated and shown in Figure 3. After screw fixation in the Latarjet procedure, the shoulder was reassessed under direct visualization using the abduction–external rotation test to confirm that no further engagement occurred.

### Arthroscopic remplissage

In the beach chair position, a pre-operative examination under anesthesia and confirmatory diagnostic arthroscopy were conducted in every patient. Hill-Sachs engagement was assessed with the arm in 90° of abduction and progressive external rotation before joint distension, and glenoid bone loss was confirmed arthroscopically through the anterosuperior portal. Both the isolated Latarjet and the Latarjet plus remplissage procedures were performed without traction.

The remplissage was performed using the technique described by Purchase et al.^17^ Anchors were placed centrally within the Hill-Sachs lesion, just lateral to the myotendinous junction of the infraspinatus. Care was taken to avoid overly medial anchor placement, which may restrict external rotation, and far-medial suture retrieval was avoided to reduce the risk of motion loss. A penetrating grasper was used to secure the posterior capsule and infraspinatus tendon, and the remplissage anchor was tied after completion of the Latarjet procedure.

**Figure 4:**
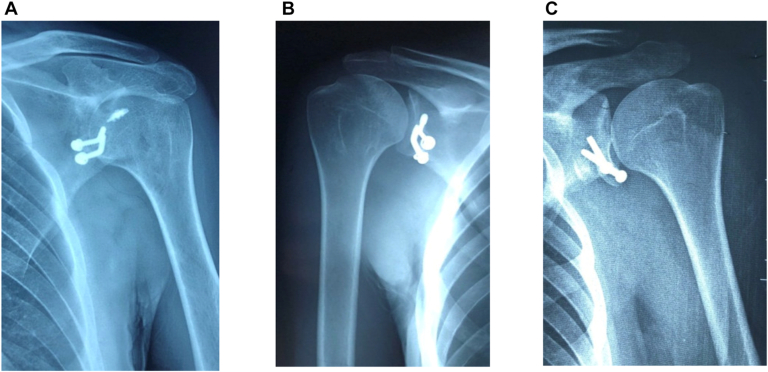
(A) Metal failure in Group 2. (B) Metal failure in group 1. (C) Metal failure in Group 1.

The congruent-arc Latarjet was performed according to the method of de Beer et al.^6^ Anthropometric parameters of the coracoid (length, width, and thickness) were evaluated as in Figure 4. After screw fixation, the shoulder was reassessed under direct visualization using the abduction–external rotation test to confirm that no further engagement occurred.

### Modified Latarjet

The modified Latarjet technique described by de Beer et al^6^ was used. A standard deltopectoral approach was performed with meticulous hemostasis, and dynamic Hohmann retractors of variable curves and sizes were utilized instead of static Steinmann pin retractors. In situ preparation of the medial aspect of the coracoid was performed and decorticated with a rasp and burr until a broad, flat cancellous surface was obtained. Before coracoid osteotomy, measurements were taken to ensure an appropriate graft length of 22-25 mm from the tip, and the osteotomy was made just anterior to the insertion of the coracoclavicular ligaments at the coracoid base. During graft dissection, the conjoint tendon insertion and the medial blood supply to the coracoid were carefully preserved.

Coracoid grafts of 22-25 mm were harvested after marking screw positions with 8-10 mm spacing. Adequate spacing between the screw holes (average 8-10 mm) and between the screw and bony edge was confirmed and marked with diathermy before drilling. During fixation, a sequential gentle tightening technique was used, as excessive force could risk screw head penetration into the coracoid graft and catastrophic fixation failure.

The shoulder was positioned in external rotation to optimize visualization of the subscapularis. The superior and inferior borders of the subscapularis were identified, with the lower limit marked by the “three sisters” of vessels. A horizontal split was made between the upper two-thirds and lower one-third of the tendon using electrocautery, with great care to avoid disinsertion from the lesser tuberosity. A transverse capsulotomy was then performed in line with the subscapularis split.

After glenoid preparation, the coracoid graft was positioned flush with the anteroinferior glenoid margin below the equator, extending the glenoid articular arc between the 3 and 5 o'clock positions (right shoulder). Intraoperative stability was confirmed after graft fixation and before tying the remplissage sutures. A horizontal capsular incision was repaired post-operatively with interrupted sutures, without additional capsulolabral repair, as labral reattachment to the defective glenoid was believed to increase joint constraint, limit ROM, and accelerate joint degeneration. Care was also taken to avoid injury to the long head of the biceps tendon during repair of the lateral-most extent of the subscapularis split.

### Rehabilitation

Passive forward flexion and abduction to 90° began on post-operative day 1. Active motion of the hand, wrist, and elbow was encouraged. The arm was immobilized in a sling for 4 weeks; resisted elbow flexion was restricted for 6 weeks. Thereafter, progressive active-assisted and active ROM was allowed. Return to contact sports and heavy labor was permitted after 6 months.

### Follow-up for groups 1 and 2

The patients were followed up at 2 weeks, 1 month, 3 months, 6 months, 12 months, and 18 months, and scores were collected at 24 months after surgery. The following steps were collected at the 24-month post-operative:

1. Visual analog pain scale
2. ROM
3. Oxford score
4. Rowe score
5. Constant score
6. Patients’ satisfaction
7. Standing X-rays anteroposterior and lateral
8. Complications

### Outcome evaluation

Clinical outcomes were assessed pre-operatively and post-operatively at 2 weeks, 1 month, 3 months, 6 months, 12 months, and at final follow-up (minimum 24 months). Outcome measures at 24 months included ROM (forward flexion, external rotation, internal rotation), Rowe score, Oxford Shoulder Instability Score-12, Constant score, visual analog scale (VAS) for pain, patient satisfaction, return to preinjury activity, and complications. Although multiple time points were collected, the 24-month outcomes were used for primary comparative analysis. Missing subjective data were obtained through telephone interviews when necessary.

### Statistical analysis

Data were analyzed using SPSS v20 (IBM Corp., Armonk, NY, USA). Continuous variables were expressed as mean ± standard deviation or median (range); categorical variables as frequency (%). Between-group comparisons used the Student’s *t*-test (continuous data) or chi-square/Fisher's exact test (categorical). Within-group pre- vs. post-operative comparisons used the paired *t*-test. Significance was set at *P* < .05 (highly significant at *P* < .01). Interobserver agreement was assessed by kappa statistics (0-0.2 = none, 0.21-0.4 = weak, 0.41-0.6 = moderate, 0.61-0.8 = good, 0.81-1.0 = excellent).

## Results

Outcomes were collected at 6 months, 12 months, and at the final follow-up. For the main analysis, we used the 24-month data. At this point, 60 shoulders in 60 patients were available (n = 30) in each group. There were no significant differences between groups regarding age (*P* = .22) or sex distribution (*P* = .30). The right side was affected in 21 patients in Group 1 and 27 patients in Group 2 (*P* = .053). The mean interval from first dislocation to surgery was 16.2 ± 12.3 months vs. 15.0 ± 9.4 months (*P* = .68). The median number of dislocations was 11 (range, 6-48) in Group 1 and 12 (range, 8-50) in Group 2, as shown in Table II.

**Table II:** Baseline characteristics between groups.

Variable	Group (I) Latarjet (n = 30)	Group (II) Latarjet plus remplissage (n = 30)	*P* value
Age (yr)	28.2 ± 5.9 (18-44)	31.1 ± 6.0 (21-44)	.06^Ns^
Sex (male/female), n	25/5	30/0	.02∗
Occupation			
Not working	0	1 (3.3%)	.11^Ns^
Heavy manual worker	15 (50.0%)	16 (53.2%)	
Employee	6 (20.0%)	6 (20.0%)	
Housewife	5 (16.7%)	0	
Player	3 (10.0%)	2 (6.6%)	
Student	1 (3.3%)	5 (16.7%)	
Smoking	15 (50.0%)	14 (46.7%)	.80^Ns^
Alcohol intake	5 (16.7%)	3 (10.0%)	.45^Ns^
Drug abuse	12 (40.0%)	7 (23.3%)	.17^Ns^
Convulsive disease	7 (23.3%)	3 (10.0%)	.16^Ns^
Affected side, right/left	21/9	27/3	.053^Ns^
Dominant side, right/left	29/1	27/3	.30^Ns^
Duration (1st dislocation–operation) (mo)	16.2 ± 12.3	15.0 ± 9.4	.68^Ns^

*NS*, nonsignificant.

∗ Significant (*P* < .05).

3D CT findings were similar between groups. No significant differences were found in GD (*P* = .19), Gd (*P* = .94), glenoid track (*P* = .27), coracoid width (*P* = .67), coracoid thickness (*P* = .93), or HSI (*P* = .39). The mean follow-up was 25.4 ± 2.4 months in Group 1 and 26.5 ± 3.5 months in Group 2 (*P* = .16) (Table III).

**Table III:** Baseline measurements, follow-up, and operative time between groups.

Variable	Group (I) Latarjet (n = 30)	Group (II) Latarjet plus remplissage (n = 30)	*P* value
Glenoid diameter (mm)	31.7 ± 1.99	30.3 ± 5.42	.19ᴺˢ
Glenoid defect (mm)	9.67 ± 1.47	9.70 ± 1.51	.94ᴺˢ
Glenoid track (mm)	16.65 ± 1.67	16.19 ± 1.52	.27ᴺˢ
HSI (mm)	24.28 ± 2.56	22.71 ± 2.75	.03∗
Coracoid width (mm)	14.67 ± 1.22	14.79 ± 0.97	.67ᴺˢ
Coracoid thickness (mm)	11.08 ± 1.03	11.06 ± 0.65	.93ᴺˢ
Follow-up (mo)	14.4 ± 2.4	17.5 ± 5.5	<.01∗
Operative time (min)	60.7 ± 12.8	86.5 ± 12.7	<.01∗

*NS*, not significant; *HSI*, Hill-Sachs interval.

∗ Significant (*P* < .05).

Pre-operative functional scores (Constant, Oxford, Rowe, and VAS) were similar between groups (all *P* > .05).

Post-operatively, all functional scores improved significantly in both groups (all *P* < .01). Although mean values varied slightly between groups, none of these differences were statistically significant: Rowe (85.67 ± 21.16 vs. 85.17 ± 17.83, *P* = .92); VAS (2.93 ± 1.86 vs. 2.73 ± 1.33, *P* = .63); Constant (84.36 ± 12.69 vs. 86.56 ± 11.41, *P* = .48); and Oxford instability score (38.70 ± 9.02 vs. 40.36 ± 9.03, *P* = .47).

Interobserver agreement for 3D CT measurements was excellent (κ = 0.88, *P* < .001). Agreement between 3D CT and anthropometric coracoid measurements was moderate (κ = 0.44, *P* < .001). “3D CT demonstrated that the Latarjet bony block effect converted 58 of 60 patients (96.7%) from off-track to on-track status, compensating for 46.70 ± 2.48% (range, 41.38-51%) of GD. In 2 patients, the coracoid was insufficient to fully restore the glenoid track. A patient belonged to Group 1 and the other belonged to Group 2 and remained clinically stable at final follow-up.

Range of motion improved significantly in both groups. In Group 1, flexion rose from 137.3° to 161.5°, external rotation from 34.7° to 59.1°, and internal rotation from 40.6° to 52.7° (all *P* < .01). In Group 2, flexion increased from 139.2° to 165.2°, external rotation from 37.0° to 45.5°, and internal rotation from 37.0° to 55.3° (all *P* < .01), as demonstrated in Table IV.

**Table IV:** Clinical outcomes between patient groups.

Variable	Group (I) Latarjet (n = 30)	Group (II) Latarjet plus remplissage (n = 30)	*P* value
Constant score			
Pre-operative	71.7 ± 6.22	72.1 ± 15.12	.89ᴺˢ
Post-operative	84.4 ± 12.69	86.6 ± 11.41	.48ᴺˢ
*P* value	<.01∗	<.01∗	
Oxford score			
Pre-operative	22.7 ± 5.40	22.5 ± 5.10	.88ᴺˢ
Post-operative	38.7 ± 9.02	40.4 ± 9.03	.47ᴺˢ
*P* value	<.01∗	<.01∗	
Rowe score			
Pre-operative	24.0 ± 6.74	25.3 ± 7.97	.50ᴺˢ
Post-operative	85.7 ± 21.16	85.2 ± 17.83	.92ᴺˢ
*P* value	<.01∗	<.01∗	
VAS			
Pre-operative	7.01 ± 1.55	7.23 ± 1.25	.55ᴺˢ
Post-operative	2.93 ± 1.86	2.73 ± 1.33	.63ᴺˢ
*P* value	<.01∗	<.01∗	
Range of motion			
Flexion			
Pre-operative	137.3 ± 17.97	139.2 ± 16.71	.67ᴺˢ
Post-operative	161.5 ± 18.24	165.2 ± 18.49	.43ᴺˢ
*P* value	<.01∗	<.01∗	
External rotation			
Pre-operative	34.7 ± 11.36	37.0 ± 17.98	.56ᴺˢ
Post-operative	59.1 ± 20.18	45.5 ± 16.29	<.01∗
*P* value	<.01∗	<.01∗	
Internal rotation			
Pre-operative	40.6 ± 8.27	37.0 ± 10.87	.15ᴺˢ
Post-operative	52.7 ± 11.12	55.3 ± 11.36	.37ᴺˢ
*P* value	<.01∗	<.01∗	
Lost flexion	14.1 ± 2.07	13.5 ± 4.68	.52ᴺˢ
Lost external rotation	12.5 ± 1.67	25.7 ± 6.0	<.01∗
Lost internal rotation	10.2 ± 3.0	9.9 ± 7.67	.84ᴺˢ
Return to preinjury activity	25 (83.3%)	26 (86.7%)	.72ᴺˢ
Satisfaction			
Very satisfied	18 (60.0%)	18 (60.0%)	.74ᴺˢ
Satisfied	7 (23.4%)	7 (23.3%)	
Neutral	1 (3.3%)	2 (6.7%)	
Dissatisfied	4 (13.3%)	2 (6.7%)	
Very dissatisfied	0	1 (3.3%)	

*NS*, not significant; *VAS*, visual analog scale.

∗ Significant (*P* < .05).

Pain levels dropped significantly after surgery in both groups. At the final follow-up, mean VAS scores were 2.93 in Group 1 and 2.73 in Group 2. The difference was not significant (*P* = .63). Overall, post-operative pain was reported in 14 of 60 patients (23.3%). Severe pain (VAS 6-9) occurred in 4 patients (2 in each group), moderate pain (VAS 4-5) in 6 patients (4 in Group 1 and 2 in Group 2), and mild pain (VAS 2-3) in 4 patients (1 in Group 1 and 3 in Group 2). Most cases were managed successfully with conservative measures, and no patients required reoperation specifically for pain.

The mean operative time was significantly longer in Group 2 (86.5 ± 12.7 min vs. 60.7 ± 12.8 min, *P* < .01), with an overall mean of 74.2 ± 18.2 min.

Overall satisfaction was high in both groups. In Group 1, 18 patients (60%) were very satisfied, 7 (23.3%) satisfied, 1 neutral, and 4 dissatisfied. In Group 2, the figures were similar: 18 (60%) very satisfied, 7 (23.3%) satisfied, 2 neutral, and 3 dissatisfied (*P* = .74).

Recurrent instability occurred in 2/30 patients (6.7%) in Group 1 and 1/30 (3.3%) in Group 2, with no significant difference.

Post-operative complications were observed in both groups without significant differences. Overall, recurrent instability occurred in 3 patients (5%). One patient in the remplissage group (3.3%) developed a brachial plexus injury affecting the upper and middle trunk, which resulted in poor functional recovery despite rehabilitation. Hardware-related issues included screw malposition, prominent screw heads, and lateralized grafts; most of these cases were managed successfully with hardware removal. Graft resorption was detected in 2 patients in the Latarjet group—one partial and asymptomatic, and 1 complete with recurrent instability requiring revision. Osteolysis around remplissage anchors was observed in 1 patient of the remplissage group, necessitating arthroscopic débridement and hardware removal. Post-operative pain was reported in 15 patients (25%), with severe pain in 4 (6.7%), moderate pain in 6 (10%), and mild pain in 5 (8.3%). Severe pain was mainly associated with recurrent instability or neurological injury, while moderate-to-mild pain was often related to hardware complications or remplissage anchors (Fig. 4). Symptomatic cases generally improved with conservative management or revision surgery (Table V).

**Table V:** Post-operative complications for each group.

Complication	Group 1 (Latarjet) (n = 30)	Group 2 (Latarjet + remplissage) (n = 30)	Total (N = 60)
Recurrent instability	2 (6.7%)	1 (3.3%)	3 (5%)
Brachial plexus injury	0	1 (3.3%)	1 (1.7%)
Screw malposition/prominent heads/lateralized grafts	Reported cases (managed with hardware removal)	Reported cases (managed with hardware removal)	Several
Graft resorption (partial/complete)	2 (1 partial, 1 complete requiring revision)	0	2 (3.3%)
Osteolysis around remplissage anchors	0	1 (requiring arthroscopic débridement + hardware removal)	1 (1.7%)
Post-operative pain (severe/moderate/mild)	2/4/1	2/2/3	4/6/4 (6.7%/10%/6.7%)

## Discussion

This study compared the outcomes of the modified Latarjet procedure alone with the modified Latarjet plus remplissage procedure in the treatment of recurrent anterior shoulder instability associated with glenoid bone loss >25% and off-track Hill-Sachs lesions. Both procedures resulted in significant improvements in functional scores, range of motion, patient satisfaction, and return to preinjury activity, with no statistically significant differences in overall outcomes.

Both groups showed some loss of external rotation, but the reduction was greater with Latarjet plus remplissage (*P* < .01). Despite being statistically significant, this difference had little clinical impact for most patients. Post-operative values for both groups overlapped within their standard deviations. Only 3 patients (1 in Group 1 and 2 in Group 2) reported functional limits due to restricted rotation. Similar to Boileau et al,^2^ our findings suggest that remplissage may predispose to external rotation limitation, though other studies have reported comparable restrictions after Bankart repair regardless of remplissage.^5^ Rehabilitation quality may also play a role in post-operative mobility.

Post-operative instability occurred in 3 patients (5%): 2 in Group 1 and 1 in Group 2. Reported causes included insufficient graft size fixed with 1 screw, uncontrolled post-operative seizures, and recurrent epileptic fits. These results are consistent with recurrence rates reported in long-term series by Hovelius et al,^12^ Mizuno et al,^15^ and systematic reviews by Griesser et al.^10^

An interesting finding of this study was that the Latarjet procedure converted 96.7% of shoulders from off-track to on-track status on post-operative 3D CT. This suggests that coracoid size may be a critical factor in restoring glenoid track coverage, and pre-operative imaging could potentially identify patients with a coracoid large enough to achieve this effect. Notably, 2 patients, 1 in each group, had insufficient coracoid dimensions to fully restore the glenoid track on post-operative 3D CT. Both remained clinically stable during follow-up, suggesting that factors other than complete track restoration may contribute to shoulder stability. Although this represents a very small subgroup, it highlights the potential value of pre-operative coracoid measurement to identify cases where remplissage or alternative augmentation might be necessary. Future studies with larger cohorts are needed to clarify whether such pre-operative planning could optimize surgical decision-making.

Complications in this study were generally procedure-related rather than technique-specific. Neurological complications occurred in 1 patient of Group 2 (3.3%) who developed a brachial plexus injury, leading to poor functional recovery despite intensive rehabilitation. Hardware-related complications included screw malposition, graft resorption, lateralized grafts, and osteolysis around anchors. Most symptomatic cases were successfully managed with hardware removal or revision surgery. Our findings align with previous reports indicating reoperation rates of 7-10% due to hardware issues.^10^^,^^11^^,^^18^

Pain was reported by 25% of patients, distributed similarly between groups. Severe pain (VAS 6-9) was found in 4 patients, 3 of whom also had recurrent instability or neurological injury. Moderate pain was related to malpositioned screws, high graft placement, or anchor osteolysis, while mild pain was often associated with remplissage anchors. Symptomatic relief was typically achieved through conservative treatment or hardware removal, consistent with previous reports.^10^

Overall, both procedures were effective in restoring stability and improving patient outcomes. Remplissage, while beneficial in addressing large Hill-Sachs lesions, was associated with longer operative times and greater loss of external rotation. Future studies should investigate whether pre-operative factors such as coracoid size predict the adequacy of Latarjet alone in converting off-track to on-track lesions, helping to individualize surgical planning.

### Limitations

The present study has several limitations. The follow-up period, although a minimum of 2 years, is relatively short for assessing long-term recurrence and graft survival. Not all patients underwent post-operative 3D CT, limiting the radiological assessment of graft remodeling. Some subjective outcome data were obtained through telephone interviews, which may introduce recall bias. Furthermore, this was a single-center study with procedures performed by a single surgeon, which may limit generalizability but also ensures technical consistency. Additionally, although external rotation was statistically different between groups, the values remained within the standard deviation range, which limits the clinical significance of this finding.

## Conclusions

Both modified Latarjet and modified Latarjet plus remplissage are reliable and effective in the treatment of recurrent anterior shoulder dislocation associated with glenoid bone loss >25% and off-track Hill-Sachs lesion. Both techniques achieved significant comparable improvement in post-operative ROM, functional instability scores, patient satisfaction, and return to pre-operative activity level. The post-operative complications were almost similar between both groups. Latarjet plus remplissage showed significant post-operative limited external rotation in comparison with the nonoperated side and a significantly longer operative time. Further larger studies with longer follow-up are warranted to support our findings.

## Disclaimers

Funding: No funding was disclosed by the authors.

Conflicts of interest: The authors, their immediate families, and any research foundations with which they are affiliated have not received any financial payments or other benefits from any commercial entity related to the subject of this article.

## Data availability statement

The datasets generated and/or analyzed during the current study are not publicly available due to patient privacy regulations but are available from the corresponding author on reasonable request.
